# Internet-based grief therapy for bereaved individuals after loss due to Haematological cancer: study protocol of a randomized controlled trial

**DOI:** 10.1186/s12888-018-1633-y

**Published:** 2018-02-27

**Authors:** Rahel Hoffmann, Julia Große, Michaela Nagl, Dietger Niederwieser, Anja Mehnert, Anette Kersting

**Affiliations:** 1 Department of Psychosomatic Medicine and Psychotherapy, University of Leipzig, Semmelweisstraße 10, 04103 Leipzig, Germany; 2 Division of Haematology and Medical Oncology, University of Leipzig, Johannisallee 32A, 04103 Leipzig, Germany; 3 Department of Medical Psychology and Medical Sociology, University Medical Centre Leipzig, Philipp-Rosenthal-Straße 55, 04103 Leipzig, Germany

**Keywords:** Bereavement, Prolonged grief, Cancer, Haematological cancer, Internet-based therapy

## Abstract

**Background:**

Approximately 10% of the individuals experiencing the death of a loved one develop prolonged grief disorder (PGD) after bereavement. Family members of haematological cancer patients might be particularly burdened since their loss experience is preceded by a very strenuous time of disease and aggressive treatment. However, support needs of relatives of cancer patients often remain unmet, also after the death of the patient. Therapeutic possibilities are enhanced by providing easily available and accessible Internet-based therapies. This study will adapt and evaluate an Internet-based grief therapy for bereaved individuals after the loss of a significant other due to haematological cancer.

**Methods:**

The efficacy of the Internet-based grief therapy is evaluated in a randomized controlled trial with a wait-list control group. Inclusion criteria are bereavement due to hematological cancer and meeting the diagnostic criteria for PGD. Exclusion criteria are severe depression, suicidality, dissociative tendency, psychosis, posttraumatic stress disorder, substance use disorder, and current psychotherapeutic or psychopharmacological treatment. The main outcome is PGD severity. Secondary outcomes are depression, anxiety, somatization, posttraumatic stress, quality of life, sleep quality, and posttraumatic growth. Data is collected pre- and posttreatment. Follow-up assessments will be conducted 3, 6, and 12 months after completion of the intervention. The Internet-based grief therapy is assumed to have at least moderate effects regarding PGD and other bereavement-related mental health outcomes. Predictors and moderators of the treatment outcome and PGD will be determined.

**Discussion:**

Individuals bereaved due to haematological cancer are at high risk for psychological distress. Tailored treatment for this particularly burdened target group is missing. Our study results will contribute to a closing of this healthcare gap.

**Trial registration:**

German Clinical Trial Register UTN: U1111–1186-6255. Registered 1 December 2016.

## Background

Grieving is an emotional reaction to the loss of a loved one and refers to the transition between the loss experience and the adaptation to it [1] whereby intense feelings of mourning and yearning are considered normal and typically decrease over time [2]. According to Stroebe and Schut [3] the process of coping with bereavement is characterized by an oscillation between loss-oriented and restoration-oriented stressors. During this process the bereaved person alternates between confrontation with and avoidance of the different tasks of grieving which results in adjustment to bereavement.

However, some individuals develop a persistent grief reaction which is described as persistent complex bereavement disorder by the DSM-5 in the chapter of diagnoses that require further research [4]. In the ICD-11 this syndrome of persistent grief will probably be included as Prolonged Grief Disorder (PGD) [5]. DSM-5 and ICD-11 criteria for grief disorders describe the same diagnostic entity, differing merely semantically [6]. PGD is characterized by intense symptoms of grief enduring for more than 6 months post-loss, separation distress, intrusive thoughts, and feelings of emptiness or meaninglessness [7]. A recent meta-analysis revealed a prevalence of about 10% for PGD among bereaved adults [8]. The loss of a loved one can not only trigger PGD but also depression, anxiety, or posttraumatic stress disorder (PTSD) [9]. Persons suffering from chronic grief experience elevated levels of depression and mortality [10].

A loss due to cancer was shown to be a risk factor for PGD [11, 12] and depression [12] and to be as distressing as an unexpected natural loss (e.g., due to cardiac arrest, accident) [12]. Cancer ranks among the leading causes of morbidity and mortality worldwide [13]. In 2013 haematological cancer was the third most common cancer-related cause of death in Germany with 18,831 people who died due to this disease [14].

Cancer is a significant psychological burden for patients and for their loved ones. During the time of illness significant others of cancer patients show high distress with prevalence rates of 20 to 46% for depression and anxiety [15, 16], and lower health-related quality of life than the general population, especially if cancer is advancing [17]. Levels of distress, depression, and anxiety of family members were shown to be similar or even higher compared to cancer patients [15, 18–20]. Declined functional status and increased physical symptoms as well as higher psychological distress in cancer patients are associated with higher caregiver distress [18, 21, 22]. Especially haematological cancer patients are burdened by long and aggressive cancer treatments [23, 24] and show high distress [19, 20, 25, 26], as do their family members [19, 20, 26, 27]. These findings suggest a particularly high risk for adverse psychological outcomes in family members of haematological cancer patients. Yet, relatives of haematological cancer patients report more unmet supportive care needs than patients [20].

In the case of bereavement caregivers of cancer patients show a deterioration in mental health [28–30]. Impaired mental health during the time of the cancer experience predicted worse mental health after bereavement [31] and PGD [32]. Caserta and colleagues argue that a burdensome time of illness may deplete resources and impede bereavement adjustment [12].

Tough relatives of haematological cancer patients may be assumed to be at heightened risk for adverse outcomes after bereavement, there is a lack of studies focusing on bereavement adjustment among relatives of patients with haematological cancer. To our knowledge, only one study examined psychological well-being after bereavement due to haematological cancer and found lower psychological well-being in bereaved parents of children who underwent haematopoietic stem cell transplantation compared to other cancer-bereaved parents [33].

These results underline the need for support for bereaved relatives of cancer patients. Easily available and accessible support can be provided by Internet-based programs [34, 35]. Compared to face-to-face therapy Internet-based interventions facilitate more flexibility and anonymity as well as faster attainability [36, 37]. Internet-based interventions and face-to-face therapy show comparable positive effects, e.g., for depression [38]. Participants in Internet-based treatments reported a positive working alliance [39–42]. Despite the advantages of Internet-based programs Northouse et al. [43] found no study with an Internet-based intervention in their review of psychosocial interventions for caregivers of cancer patients. Therefore, our Internet-based grief therapy constitutes an important innovation, closing a research and supply gap.

In our study we use an Internet-based cognitive-behavioural grief therapy originally developed as “Interapy” by Lange and colleagues for the treatment of posttraumatic stress [35]. For the treatment of PGD cognitive-behavioural therapy proved efficacious, particularly exposure therapy [44, 45]. Interapy was adapted for PGD [46] and its efficacy was shown for various groups of bereaved individuals showing medium to large treatment effects [46, 47].

The main goal of our study is the adaptation and evaluation of the Internet-based cognitive-behavioural grief therapy for bereaved persons after the loss of a significant other due to haematological cancer, targeting primarily the reduction of PGD severity. The results of our study will provide information about the efficacy of Internet-based therapy for people who experienced a loss which is usually expected but preceded by a very burdensome time of disease and aggressive treatment.

## Methods

The guided text-based intervention for people who suffer from PGD after bereavement due to haematological cancer is currently evaluated in a randomized waitlist-controlled trial. Questionnaires are administered at screening for eligibility (T-1), at baseline (T0), during the intervention (monitoring), at post-treatment (T1) and at three follow-up points (T2–4; 3, 6, and 12 months after intervention completion). Severity of PGD symptoms as measured with the Inventory of Complicated Grief (ICG) [48] is the main outcome. All questionnaires and the intervention are administered via a secure website and data is stored on secure storage devices. The procedure is described in detail below.

### Procedure

#### Recruitment practices

Short information about the study and a link to the study website is sent to a multitude of stakeholders, including support groups, charities, insurance companies, clinics and medical practices, owners or contact persons of relevant websites, online communities, and blogs in Germany. Flyers are sent via mail by request and distributed in departments of the University Medical Centre Leipzig, e.g., Psychosomatic Medicine, Medical Psychology and Medical Sociology, and Haematology and Medical Oncology.

The study website provides thorough information about the study and the Internet-based grief therapy. Interested persons can apply by submitting a screening questionnaire which determines whether they fulfil eligibility criteria. Contact information of the research team is provided to be addressed in case of questions and remarks.

#### Participant timeline

**Fig. 1:**
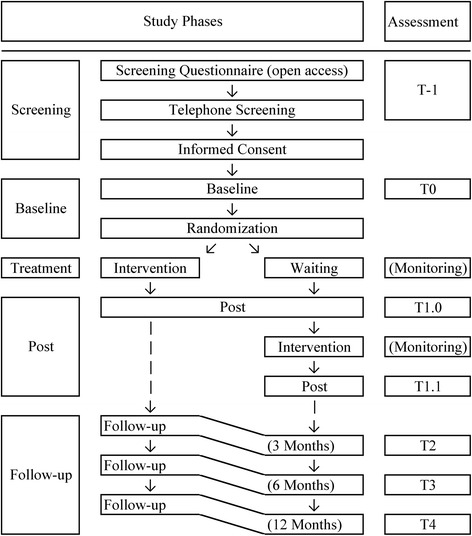
Participant timeline

The procedure from screening to follow-up assessments is depicted in Fig. 1.

*T-1, screening:* Participants apply for the study via an openly accessible online questionnaire. Participants who may fulfil the eligibility criteria as described below will be contacted for a telephone screening, which includes the Prolonged Grief Interview [7, 49] and in case of eligibility concerns queries regarding participant’s answers in the online questionnaire. Participants who do not fulfil the eligibility criteria but show signs of suicidal ideation will also be called to ensure their safety and provide support in finding immediate help if necessary. Participants who are excluded from the study after the screening process will receive information about the reasons via e-mail and be offered help in finding an alternative treatment.

*Informed consent:* Participants meeting the eligibility criteria will be sent thorough information on the study and asked to send back a consent form, which also includes contact information of the participant’s general practitioner, who will be contacted in case of endangerment to self or others. Participants are informed about this requirement. Questions can be addressed to the research team at any time.

*T0, baseline:* After written informed consent, participants receive a personal link to the baseline questionnaire. Upon submission of this questionnaire participants are randomized as described below.

*Treatment:* The intervention group (IG) receives the intervention as described below. Those assigned to the waitlist-control group (WCG) will wait for five weeks before completing the first post-treatment assessment (T1.0). Subsequently, the same intervention as described above will be provided and an identical post-treatment assessment will be administered (T1.1) after the intervention. Participants in the WCG will be informed about this procedure immediately after randomization. For the sake of clarity the following nomenclature will be used subsequently: “treatment” describes the study phase between baseline and post-measurement and includes IG and WCG; “intervention” describes the Internet-based therapy that is conducted during treatment for IG and after the first post-treatment assessment for WCG.

*T1, post-treatment:* After completion of treatment, participants receive a link to the post-treatment questionnaire. The WCG receives an identical questionnaire again after completing the intervention.

*T2–4, follow-up:* 3, 6, and 12 months after cessation of the intervention, links to online follow-up questionnaires are sent to participants of both groups. All follow-up questionnaires are identical.

#### Randomization

Randomization takes place after the baseline assessment. Participants are randomized into one of two groups: IG or WCG. A permutated block randomization with a block size of four and equal probabilities to be sampled into either group is carried out with pseudo-seeds, using MersenneTwister. The used software was “Randomization in Treatment Arms” (RITA). Neither participants, nor the research team were blind to group allocation. Yet, this is not expected to lead to biased results, since all assessments after randomization are carried out anonymously and automated via online questionnaires.

### Participants

#### Sample size and power calculation

Previous studies found effects of the Internet-based grief therapy of at least moderate size [46, 47]. Assuming moderate effect sizes, an alpha level of 0.05, and statistical power of 80% a target sample of 128 participants (64 for each group) is intended.

#### Eligibility criteria

Participants are eligible for the study if they are 18 years or older, speak German, have Internet access and meet the diagnostic criteria for PGD after bereavement due to haematological cancer. Exclusion criteria are current psychotherapy or unstable psychopharmacological treatment with changes within the last 6 weeks, severe depression, suicide ideation, dissociative tendency, psychosis, PTSD due to an event other than the loss, substance use disorder, and cognitive or physical impairments which make treatment participation impossible.

### Intervention

Participants will receive therapist-assisted Internet-based grief therapy which applies the paradigm of structured writing. All therapeutic content, such as general information and writing instructions, is presented through a secure website (“Beranet”). Communication with the therapist is also conducted via an e-mail function of this website.

The Internet-based cognitive-behavioural grief therapy aims at working through the grief as well as coping with the new situation [46]. It is derived from a rationale developed by Lange et al. for individuals suffering from posttraumatic stress [35] which was adapted for PGD by Wagner et al. [46, 50, 51]. It is structured as a sequence of ten writing tasks in three phases: (1) self-confrontation, (2) cognitive restructuring, and (3) social sharing. The first phase focuses on loss-oriented coping, whereas phases two and three refer to restoration and integration of the loss experience [46].

Participants are instructed to plan out two writing tasks per week in advance, each lasting 45 min. They receive access to each writing task upon having completed the previous task. Once a week participants receive thorough feedback for their writing tasks from their therapist. All therapists are psychologists who were trained in the application of the intervention manual and receive supervision. Instructions for the writing tasks are mainly standardized and therapist instructions for individualized feedback are highly structured. Other than sending new writing tasks and feedback, therapists engage in further communication when directly contacted by the participant or when participants express critical experiences such as high distress or suicidal thoughts. In this case therapists address existing issues via mail or, if necessary, telephone. In case of endangerment of self or others, the participant’s general practitioner will be contacted to initiate immediate care for the participant.

Prior to the first phase, participants receive general information on the treatment, psychoeducation on the phases, and instructions on how to use the treatment platform. An overview of the Internet-based grief therapy and monitoring can be found in Table 1.

**Table 1:** Intervention overview

Week	Phase	Procedure				
		Pre-Task- Monitoring		Task	Post-Task Monitoring	
1	Phase 1: Confrontation	SAM		Task 1	SAM	
		SAM	PHQ-9	Task 2	SAM	
		therapist feedback				
2		SAM		Task 3	SAM	
		SAM	PHQ-9	Task 4	SAM	WAI-S
		therapist feedback				
3	Phase 2: Cognitive reappraisal	SAM		Task 5	SAM	
		SAM	PHQ-9	Task 6	SAM	
		therapist feedback				
4		SAM		Task 7	SAM	
		SAM	PHQ-9	Task 8	SAM	WAI-S
		therapist feedback				
5	Phase 3: Social Sharing	SAM		Task 9	SAM	
		SAM	PHQ-9	Task 10	SAM	WAI-S
		therapist feedback				

*SAM*, Self-Assessment-Manikin; *PHQ-9*, Patient Health Questionnaire; *WAI-S*, Working Alliance Inventory

*Phase 1: Self-confrontation.* In four writing tasks participants describe their loss experience with a special emphasis on emotional and cognitive processes. They are instructed to write in as much detail as possible focusing on emotional and sensory perceptions, use present tense and first person, and not mind possible issues of style, grammar or orthography. The goal of this phase is to weaken feelings such as fear and guilt through reprocessing and therefore reduce avoidant behaviour.

*Phase 2: Cognitive reappraisal.* The next four writing tasks focus on a change of perspective to help participants develop realistic and helpful coping strategies. This is achieved by instructing participants to compose a supportive letter to a (possibly hypothetical) friend who endured the same kind of loss. The letter should reflect on and acknowledge burdensome feelings like guilt, fear, or anger, but also provide correction of unrealistic assumptions and dysfunctional thoughts. Participants are instructed to encourage their friend in activating resources, such as positive activities or social contacts as well as in finding rituals to express their mourning. The goal of this phase is to help participants define a new role for themselves and regain a sense of control over their lives.

*Phase 3: Social Sharing.* In the last two writing tasks participants are instructed to write a letter to a person concerned with the loss who can be also themselves or the deceased. The last letter serves as an opportunity to summarize and communicate what they may have learned during the therapeutic process and what they want to implement to cope with their loss experience.

New writing tasks are only released if the previous task has been completed. Participants can access past instructions, their texts and therapist feedback throughout the intervention and are encouraged to download all material for later rereading. Support for technical issues and issues regarding the intervention itself is provided via e-mail or telephone if necessary.

Before and after each writing task, participants complete a monitoring (Table 1) consisting of Self-Assessment-Manikin (SAM [52]), Patient Health Questionnaire (PHQ-9 [53]), and Working Alliance Inventory (WAI-S [54]).

### Measurements

The ICG [48] is used in its German version [55] to measure the primary outcome severity of PGD symptoms. It measures severity of PGD by assessing symptoms related to grief like yearning, intrusive thoughts, or resentment regarding the loss in 19 items which are rated by participants on a five-point Likert scale (never-always, 0–4) with regard to the last month. Three additional items were administered that are not included in the sum score but shall serve future comparability in the case of inclusion of PGD in ICD-11 as suggested by Maercker et al. [5]. They address feelings of guilt, difficulty accessing positive memories and anhedonia in questions adapted from Xiu et al. [56] as follows “I feel guilty about mistakes I made with regard to his/her death”, “It is really difficult for me to remember in detail happy moments with or images of him/her from the times before he/she died,” and “I no longer feel able to experience happiness, contentment, or joy since the loss of this person.”

Secondary outcomes, screening variables, moderators and mediators as well as used measures are summarized in Table 2.

**Table 2:** Measurement tools

Construct	Instrument (abbreviation) [original and German source], additional information		Rating		Reliability	T-1	T0	Monitoring	T1-T4
		Item No.	Likert scale wording (scores)	Time frame					
Prolonged grief	Inventory of Complicated Grief (ICG) [48, 55], Cut-off^a^: ≥25 (sum score) [48]	19	never-always (0–4)	last month	*α*=.87, r_tt_ = .69 [55]	x	x		x
	Additional items [56], German version received from A. Maercker	3	never-always (0–4)		–	x	x		x
	Prolonged Grief-13 – Interview version (IKT), [49, 61]	13	various formats (mostly 5-pointlLikert scales)	last month	–	x			
Depression	Patient Health Questionnaire (PHQ-9) [53, 62], Cut-off: ≥20 (sum, for severe depression) [63]	9	not at all-nearly every day (0–3)	last 2 weeks	*α*=.88 [64]	x	x	x	x
Posttraumatic stress	Impact of Event Scale-Revised (IES-R) [65, 66], Cut-off: > 0 (regression formula) [66], Screening: due to an event other than the loss, Baseline and post-assessments: due to loss	22	not at all-often (0,1,3,5)	last week	*α*=.71–.90, r_tt_ = .66–.80 [66]	x	x		x
Suicidal Ideation	Scale for Suicide Ideation (BSS or BSIS) [67, 68]	21	various formats (3 nuances each)	last week	*α*=.84–.89 [69]	x			
Psychosis	Dutch Screening Device for Psychotic Disorder (SDPD) [57], own translation from previous project, Cut-off: ≥13 (sum) [57]	8	not at all-completely true (1–5)	last 5 years	*α*=.68–.86 [57]	x			
Dissociation	Somatoform Dissociation Questionnaire (SDQ-5) [70, 71], Cut-off: ≥8 (sum) [70]	5	not applicable-highly applicable (1–5)	past year	*α*=.91, r_tt_ = .89 (long form) [71]	x			
Somatization	Patient Health Questionnaire (PHQ-15) [53, 62]	15	not bothered at all-bothered a lot (0–2)	last 4 weeks	*α*=.79 [64]		x		x
Anxiety	Generalized Anxiety Disorder Screener (GAD-7) [72, 73]	7	not at all-nearly every day (0–3)	last 2 weeks	*α*=.89 [73]		x		x
Health-related quality of life	Short-Form Health Survey (SF-12) [74, 75]	12	various formats	last 4 weeks	*α*=.57–.94 [75]		x		x
Sleep quality	Pittsburgh Sleep Quality Index (PSQI) [76, 77]	10	various formats	last 4 weeks	*α*=.85, r_tt_ = .87 [77]		x		x
Posttraumatic Growth	Posttraumatic Growth Inventory (PGI) [78, 79]	21	not at all-to a very great degree (0–5)	present	*α*=.92 [79]		x		x
Avoidance	Depressive and Anxious Avoidance in Prolonged Grief Questionnaire (DAAPGQ) [80], own translation	9	not at all true-completely true (0–7)	last month	*α*=.74–.90 [80]		x		x
Religiousness	Systems of Belief Inventory (SBI-15R) [81, 82]	15	completely true-not at all true (1–4)	present	*α*>.87 [82]		x		
Separation anxiety	Adult Separation Anxiety Questionnaire (ASA-27) [58], own translation	27	this has never happened-this happens very often (0–3)	lifetime	*α*=.95 [58]		x		
Attachment style	Relationships Questionnaire (RQ) [83, 84]	4	disagree strongly-agree strongly (1–7)	present/lifetime	–		x		
Quality of relationship to the deceased	Quality of Relationships Inventory (QRI) [85, 86] – adapted	25	not true-almost always true (1–4)	time before loss	*α*=.82–.89 [86]		x		
Childhood abuse and neglect	Childhood Trauma Questionnaire (CTQ) [87, 88]	28	never-very often (1–5)	childhood	*α*=.55–.89 [89]		x		
Circumstances surrounding the death, preparedness	Perception of circumstances surrounding the death and preparedness [59], own translation	4	various formats (7 nuances each)	time of loss	–		x		
Social support	Berlin Social Support Sales (BSSS) [90] (originally German), recipient version	32	strongly disagree-strongly agree (1–4)	present	*α*=.63–.83 [91]		x		x
Dependency	Depressive Experience Questionnaire (DEQ) dependency subscale [92, 93]	26	strongly disagree-strongly agree (1–7)	present	r_tt_ = .75 [93]		x		x
Self-esteem	Rosenberg self-esteem scale [94, 95]	10	not at all true-completely true (0–3)	present	*α*=.72–.85 [95]		x		x
Self-efficacy	Skala zur Allgemeinen Selbstwirksamkeitserwartung (Self-Efficacy Scale, SWE) [96] (originally German)	10	not at all true-completely true (1–4)	present	*α*=.80–.90 [96]		x		x
Coping strategies	Brief COPE [97, 98]	28	not at all-a lot (1–4)	lifetime	*α*=.61–.81 [98]		x		x
Stigma	Grief Experience Questionnaire (GEQ), subscale Stigmatization [60], own translation	10	never-almost always (1–5)	since loss	*α*=.86 (English version) [60]		x		x
Working alliance	Working Alliance Inventory Short Form (WAI-S) [54, 99] (only Monitoring and T1)	12	never-always (1–7)	intervention	*α*=.81–.91 [99]			x	x
Mood	Self-Assessment Manikin (SAM) [52]	3	(1–9)	present	–			x	

^a^Where a cut-off value is provided, it is used at T-1 to determine whether a participant fulfils eligibility criteria

Abbreviations: *α*=Chronbach’s alpha (internal consistency); r_tt_ = test-retest reliability

Published German translations of measurement tools are used where available. For all other measurement tools [57–60], own translations were achieved as follows: the first authors translated the measurement tools from English to German. A native English speaker retranslated the result, which was then checked for accordance with the original tool. Any inconsistencies and challenges in translating, e.g., idioms, were discussed thoroughly within the research team.

In addition to the variables listed in Table 2, sociodemographic variables, current medical problems, drug and alcohol consumption, history of previous losses, traumatic experiences, and of psychological problems, and help-seeking behaviour are assessed at baseline (T0).

All measures except the Prolonged Grief Interview [49, 61] are assessed by online self-assessment questionnaires which minimizes potential assessment bias.

### Statistical analysis

Demographic data and main outcomes will be reported using descriptive statistics. Chi-square and t-tests will be performed to examine whether randomization resulted in comparable groups and whether selective dropout occurred with regard to any pre-treatment characteristics.

To test the treatment effect, i.e. a significantly greater decrease in PGD and other mental health outcomes from baseline to post-treatment in the IG than in the WCG, a 2 × 2 repeated measure analyses of variance (ANOVA) will be conducted with time as the within-subject factor (baseline vs. post-treatment) and group as the between subject-factor (IG vs. WCG). Stability of treatment effects at 3, 6, and 12 month follow-up will be tested using two-tailed t-tests. Cohen’s d will be calculated to present effect sizes. Results will be shown for each outcome measure. Intention-to-treat analyses and completer analyses will be provided. Predictors of improvement in outcome measures and of dropout will be determined with linear and logistic regression analyses. To identify potential risk and protective factors for PGD severity and other bereavement outcomes as our secondary aim we will perform hierarchical regression analyses with baseline data, e.g., with religiousness, coping strategies, and attachment style as independent variables. All analyses will be conducted using SPSS, with an alpha level of 0.05.

## Discussion

Family members of haematological cancer patients are highly burdened since they face high cancer-related distress which continues beyond bereavement. Their support needs often remain unmet. Easily available and accessible support is provided by Internet-based treatment programmes which were shown to have similar positive effects as face-to-face therapy, e.g., for depression [38]. To our knowledge there are no guided Internet-based therapies for bereaved individuals after the loss of a loved one due to haematological cancer. Our study aims at adapting and evaluating an Internet-based cognitive-behavioural grief therapy for this target group. Results of the study will provide information about the applicability and short- and long-term efficacy of the treatment regarding bereavement due to haematological cancer.

## Abbreviations

ANOVA: analysis of variance
ASA-27: Adult Separation Anxiety Questionnaire
BSS, BSIS: Scale for Suicide Ideation
BSSS: Berlin Social Support Sales
CTQ: Childhood Trauma Questionnaire
DAAPGQ: Depressive and Anxious Avoidance in Prolonged Grief Questionnaire
DEQ: Depressive Experience Questionnaire
DSM-V: Diagnostic and Statistical Manual of Mental Disorders
GAD-7: Generalized Anxiety Disorder Screener
GEQ: Grief Experience Questionnaire
ICD-11: International Statistical Classification of Diseases and Related Health Problems
ICG: Inventory of Complicated Grief
IES-R: Impact of Event Scale-Revised
IG: intervention group
IKT: Prolonged Grief-13 – Interview version
PGD: prolonged grief disorder
PGI: Posttraumatic Growth Inventory
PHQ: Patient Health Questionnaire
PSQI: Pittsburgh Sleep Quality Index
PTSD: posttraumatic stress disorder
QRI: Quality of Relationships Inventory
RQ: Relationships Questionnaire
SAM: Self-Assessment-Manikin
SBI-15R: Systems of Belief Inventory
SDPD: Dutch Screening Device for Psychotic Disorder
SDQ-5: Somatoform Dissociation Questionnaire
SF-12: Short-Form Health Survey
SPSS: Statistical Package for the Social Sciences
SWE: Self-Efficacy Scale
WAI: Working Alliance Inventory
WCG: waitlist-control group
